# Crystal structure of ethyl 8-chloro-4-oxo-1,4-di­hydro­quinoline-3-carboxyl­ate

**DOI:** 10.1107/S2056989015013171

**Published:** 2015-07-15

**Authors:** Yoshinobu Ishikawa, Nanako Yoshida

**Affiliations:** a School of Pharmaceutical Sciences, University of Shizuoka, 52-1 Yada, Suruga-ku, Shizuoka 422-8526 , Japan

**Keywords:** crystal structure, quinolone, hydrogen bonding

## Abstract

In the title compound, C_12_H_10_ClNO_3_, the asymmetric unit comprises two independent mol­ecules, and the dihedral angle between the least-square planes of the quinoline ring systems of these mol­ecules is 73.30 (5)°. In the crystal, N—H⋯O hydrogen bonds between the independent mol­ecules lead to supra­molecular layers parallel to (-1-10); both N—H H atoms are bifurcated.

## Related literature   

For the biological background of this study, see: Mugnaini *et al.* (2009 ▸); Ishikawa & Fujii (2011 ▸); Bisacchi (2015 ▸). For the synthesis of the title compound, see: Ozeki *et al.* (1987 ▸). For related structures, see: Garudachari *et al.* (2012 ▸, 2013 ▸); Ishikawa & Yoshida (2014 ▸).
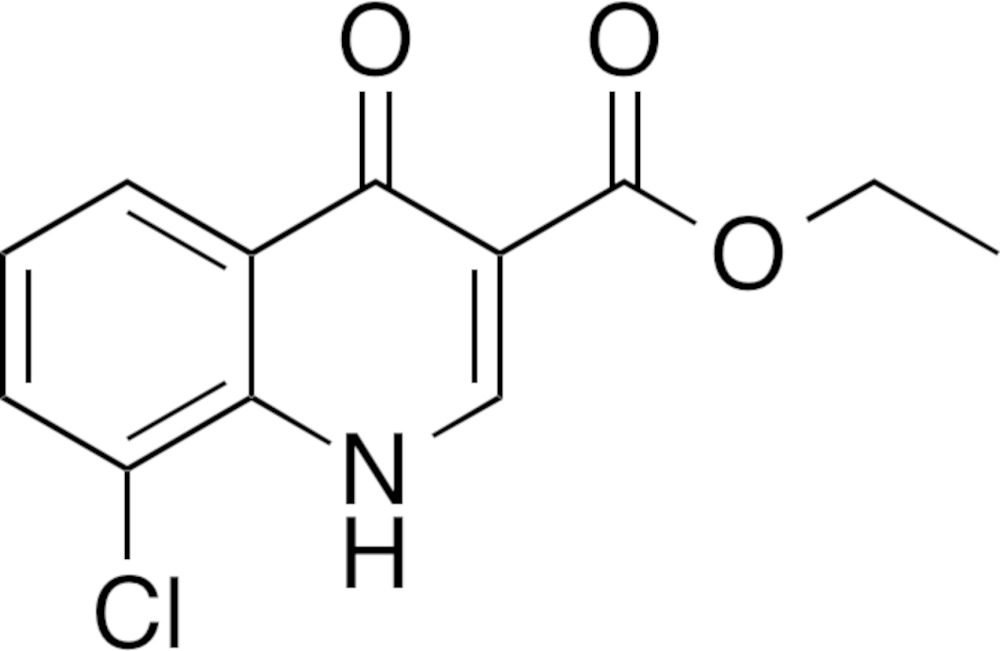


## Experimental   

### Crystal data  

C_12_H_10_ClNO_3_
*M* *_r_* = 251.67
Triclinic, 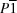

*a* = 9.328 (5) Å
*b* = 11.043 (2) Å
*c* = 12.350 (4) Å
α = 73.298 (17)°
β = 70.57 (3)°
γ = 77.22 (3)°
*V* = 1137.8 (7) Å^3^
*Z* = 4
Cu *K*α radiation
μ = 2.96 mm^−1^
*T* = 298 K
0.25 × 0.15 × 0.15 mm

### Data collection  

Rigaku AFC7R diffractometer
Absorption correction: ψ scan (North *et al.*, 1968 ▸) *T*_min_ = 0.436, *T*_max_ = 0.642
5652 measured reflections
4147 independent reflections
3328 reflections with *F* ^2^ > 2.0σ(*F* ^2^)
*R*_int_ = 0.047
3 standard reflections every 150 reflections intensity decay: −0.9%

### Refinement  

*R*[*F* ^2^ > 2σ(*F* ^2^)] = 0.066
*wR*(*F* ^2^) = 0.184
*S* = 1.05
4147 reflections
309 parameters
H-atom parameters constrained
Δρ_max_ = 0.49 e Å^−3^
Δρ_min_ = −0.41 e Å^−3^

### 

Data collection: *WinAFC* (Rigaku, 1999 ▸); cell refinement: *WinAFC*; data reduction: *WinAFC*; program(s) used to solve structure: *SIR92* (Altomare *et al.*, 1994 ▸); program(s) used to refine structure: *SHELXL97* (Sheldrick, 2008 ▸); molecular graphics: *CrystalStructure* (Rigaku, 2010 ▸); software used to prepare material for publication: *CrystalStructure*.

## Supplementary Material

Crystal structure: contains datablock(s) General, I. DOI: 10.1107/S2056989015013171/tk5374sup1.cif

Structure factors: contains datablock(s) I. DOI: 10.1107/S2056989015013171/tk5374Isup2.hkl

Supporting information file. DOI: 10.1107/S2056989015013171/tk5374Isup3.cml

**Fig. 1.**
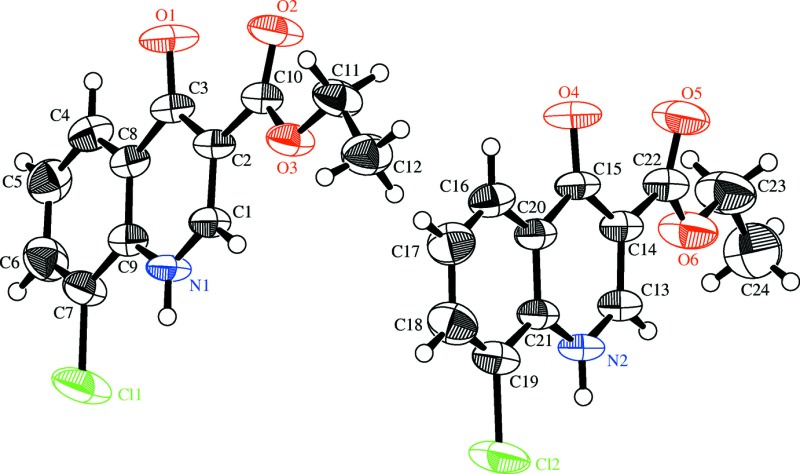
The molecular structure of the title compound, with displacement ellipsoids drawn at the 50% probability level. Hydrogen atoms are shown as small spheres of arbitrary radius.

. DOI: 10.1107/S2056989015013171/tk5374fig1.tif
The mol­ecular structure of the title compound, with displacement ellipsoids drawn at the 50% probability level. Hydrogen atoms are shown as small spheres of arbitrary radius.

**Fig. 2.**
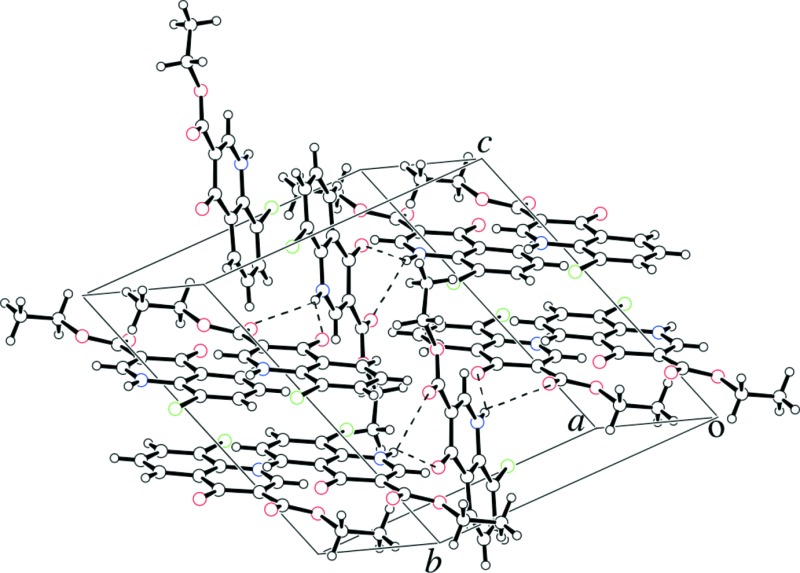
A crystal packing view of the title compound. Intermolecular hydrogen bonds are represented as dashed lines.

. DOI: 10.1107/S2056989015013171/tk5374fig2.tif
A crystal packing view of the title compound. Inter­molecular hydrogen bonds are represented as dashed lines.

CCDC reference: 1411658

Additional supporting information:  crystallographic information; 3D view; checkCIF report

## Figures and Tables

**Table 1:** Hydrogen-bond geometry (, )

*D*H*A*	*D*H	H*A*	*D* *A*	*D*H*A*
N1H1*A*O4^i^	0.86	1.98	2.748(4)	148
N1H1*A*O5^i^	0.86	2.51	3.035(4)	121
N2H2O1^ii^	0.86	2.04	2.777(4)	144
N2H2O2^ii^	0.86	2.48	3.064(4)	126

Symmetry codes: (i) 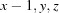; (ii) 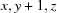.

## supplementary crystallographic information

### S1. Comment

4-Quinolones show inhibition not only to Gram negative and Gram positive bacteria (Bisacchi, 2015), but also to human immunodeficiency virus (HIV) (Mugnaini *et al.*, 2009). The inhibition to HIV is derived from their chelating ability to metal ions in the active site of metalloenzyme HIV integrase. According to our inhibitor design targeting metalloenzyme influenza virus RNA polymerase (Ishikawa & Fujii, 2011), we synthesized the title compound as a synthetic intermediate of final products.

The asymmetric unit contains two independent molecules, as shown in Fig. 1. The dihedral angle between the least-square planes of the quinoline rings of the 4-quinolone units is 73.30 (5)°. In the crystal, face-to-face π-π stacking interactions are found between the molecules and their inversion-symmetry equivalents^i,ii^ [centroid–centroid distances between the benzene rings of the 4-quinolone units = 3.597 (3)^i^ and 3.881 (3) Å^ii^, i: –*x* + 1, –*y* + 1, –*z* + 2, ii: –*x* + 2, –*y* + 2, –*z* + 1]. Molecules A are further linked with the translation-symmetry equivalents of the molecules B through bidentate N–H···O hydrogen bonds, as shown in Fig. 2.

### S2. Experimental

The title compound was synthesized according to the literature (Ozeki *et al*. 1987). Single crystals suitable for X-ray diffraction were obtained by slow evaporation of an *N*,*N*-dimethylforamide solution of the compound at room temperature.

### S3. Refinement

The H atoms of secondary amine [N—H 0.86 Å, *U*_iso_(H) = 1.2*U*_eq_(N)], methylene [C—H = 0.97 Å, *U*_iso_(H) = 1.2*U*_eq_(C)], and phenyl groups [C—H 0.93 Å, *U*_iso_(H) = 1.2*U*_eq_(C)] were placed in their geometric positions, and refined using a riding model. A rotating group model was applied for the H atoms of the methyl groups [C—H = 0.96 Å, *U*_iso_(H) = 1.2*U*_eq_(C)].

### Crystal data

**Table:**

C_12_H_10_ClNO_3_	*Z* = 4
*M**_r_* = 251.67	*F*(000) = 520.00
Triclinic, *P*1	*D*_x_ = 1.469 Mg m^−^^3^
Hall symbol: -P 1	Cu *K*α radiation, λ = 1.54178 Å
*a* = 9.328 (5) Å	Cell parameters from 25 reflections
*b* = 11.043 (2) Å	θ = 25.0–29.3°
*c* = 12.350 (4) Å	µ = 2.96 mm^−^^1^
α = 73.298 (17)°	*T* = 298 K
β = 70.57 (3)°	Prismatic, colorless
γ = 77.22 (3)°	0.25 × 0.15 × 0.15 mm
*V* = 1137.8 (7) Å^3^	

### Data collection

**Table:**

Rigaku AFC7R diffractometer	*R*_int_ = 0.047
ω–2θ scans	θ_max_ = 68.0°
Absorption correction: ψ scan (North *et al.*, 1968)	*h* = −10→11
*T*_min_ = 0.436, *T*_max_ = 0.642	*k* = −9→13
5652 measured reflections	*l* = −14→14
4147 independent reflections	3 standard reflections every 150 reflections
3328 reflections with *F*^2^ > 2.0σ(*F*^2^)	intensity decay: −0.9%

### Refinement

**Table:**

Refinement on *F*^2^	Secondary atom site location: difference Fourier map
*R*[*F*^2^ > 2σ(*F*^2^)] = 0.066	Hydrogen site location: inferred from neighbouring sites
*wR*(*F*^2^) = 0.184	H-atom parameters constrained
*S* = 1.05	*w* = 1/[σ^2^(*F*_o_^2^) + (0.1039*P*)^2^ + 0.6522*P*] where *P* = (*F*_o_^2^ + 2*F*_c_^2^)/3
4147 reflections	(Δ/σ)_max_ < 0.001
309 parameters	Δρ_max_ = 0.49 e Å^−^^3^
0 restraints	Δρ_min_ = −0.41 e Å^−^^3^
Primary atom site location: structure-invariant direct methods	

### Special details

**Table:**

Refinement. Refinement was performed using all reflections. The weighted *R*-factor (*wR*) and goodness of fit (*S*) are based on *F*^2^. *R*-factor (gt) are based on *F*. The threshold expression of *F*^2^ > 2.0 σ(*F*^2^) is used only for calculating *R*-factor (gt).

### Fractional atomic coordinates and isotropic or equivalent isotropic displacement parameters (Å^2^)

**Table:**

	*x*	*y*	*z*	*U*_iso_*/*U*_eq_	
Cl1	0.08408 (10)	0.65841 (10)	0.98364 (8)	0.0840 (4)	
Cl2	0.58312 (10)	1.16556 (10)	0.62449 (10)	0.0843 (4)	
O1	0.6848 (3)	0.2777 (2)	0.82920 (19)	0.0660 (7)	
O2	0.8378 (3)	0.3772 (3)	0.5944 (2)	0.0701 (7)	
O3	0.7027 (3)	0.5425 (2)	0.50191 (17)	0.0557 (6)	
O4	1.1706 (3)	0.7698 (2)	0.6817 (2)	0.0641 (7)	
O5	1.3355 (3)	0.8720 (3)	0.7792 (3)	0.0807 (8)	
O6	1.1962 (3)	1.0279 (3)	0.8650 (3)	0.0758 (8)	
N1	0.3633 (3)	0.5894 (2)	0.7925 (2)	0.0463 (6)	
N2	0.8636 (3)	1.0913 (2)	0.7116 (2)	0.0498 (6)	
C1	0.4819 (3)	0.5701 (3)	0.6996 (3)	0.0439 (6)	
C2	0.5972 (3)	0.4684 (3)	0.7047 (3)	0.0433 (6)	
C3	0.5924 (4)	0.3757 (3)	0.8155 (3)	0.0446 (6)	
C4	0.4510 (4)	0.3239 (3)	1.0303 (3)	0.0537 (7)	
C5	0.3293 (4)	0.3460 (4)	1.1246 (3)	0.0602 (8)	
C6	0.2151 (4)	0.4483 (4)	1.1103 (3)	0.0631 (9)	
C7	0.2249 (4)	0.5289 (3)	1.0010 (3)	0.0531 (7)	
C8	0.4622 (3)	0.4037 (3)	0.9171 (3)	0.0433 (6)	
C9	0.3498 (3)	0.5084 (3)	0.9022 (3)	0.0429 (6)	
C10	0.7249 (3)	0.4555 (3)	0.5978 (3)	0.0455 (6)	
C11	0.8225 (4)	0.5353 (4)	0.3913 (3)	0.0622 (9)	
C12	0.7820 (5)	0.6430 (4)	0.2991 (3)	0.0687 (9)	
C13	0.9825 (4)	1.0692 (3)	0.7539 (3)	0.0490 (7)	
C14	1.0937 (4)	0.9640 (3)	0.7463 (3)	0.0472 (7)	
C15	1.0809 (4)	0.8692 (3)	0.6907 (3)	0.0467 (7)	
C16	0.9258 (4)	0.8116 (3)	0.5878 (3)	0.0522 (7)	
C17	0.8017 (4)	0.8339 (4)	0.5473 (3)	0.0615 (8)	
C18	0.6941 (4)	0.9426 (4)	0.5603 (4)	0.0662 (9)	
C19	0.7149 (4)	1.0284 (3)	0.6131 (3)	0.0559 (8)	
C20	0.9476 (3)	0.8971 (3)	0.6448 (3)	0.0446 (6)	
C21	0.8410 (3)	1.0076 (3)	0.6566 (3)	0.0444 (6)	
C22	1.2215 (4)	0.9479 (3)	0.7954 (3)	0.0544 (7)	
C23	1.3084 (5)	1.0104 (5)	0.9282 (5)	0.0885 (13)	
C24	1.2729 (6)	1.1118 (5)	0.9879 (5)	0.0966 (14)	
H1	0.4869	0.6285	0.6276	0.0527*	
H1A	0.2936	0.6539	0.7837	0.0555*	
H2	0.7989	1.1595	0.7187	0.0597*	
H4	0.5272	0.2552	1.0411	0.0645*	
H5	0.3230	0.2921	1.1988	0.0723*	
H6	0.1319	0.4623	1.1747	0.0757*	
H11A	0.8290	0.4551	0.3713	0.0747*	
H11B	0.9212	0.5406	0.3986	0.0747*	
H12A	0.7703	0.7216	0.3219	0.0824*	
H12B	0.6873	0.6341	0.2893	0.0824*	
H12C	0.8619	0.6438	0.2260	0.0824*	
H13	0.9916	1.1284	0.7909	0.0588*	
H16	0.9973	0.7392	0.5779	0.0626*	
H17	0.7882	0.7763	0.5106	0.0738*	
H18	0.6086	0.9569	0.5333	0.0794*	
H23A	1.3054	0.9287	0.9850	0.1062*	
H23B	1.4107	1.0114	0.8733	0.1062*	
H24A	1.1696	1.1130	1.0390	0.1159*	
H24B	1.2828	1.1918	0.9308	0.1159*	
H24C	1.3426	1.0985	1.0338	0.1159*	

### Atomic displacement parameters (Å^2^)

**Table:**

	*U*^11^	*U*^22^	*U*^33^	*U*^12^	*U*^13^	*U*^23^
Cl1	0.0547 (5)	0.0919 (7)	0.0693 (6)	0.0301 (5)	0.0002 (4)	−0.0157 (5)
Cl2	0.0593 (6)	0.0814 (7)	0.1179 (8)	0.0357 (5)	−0.0449 (6)	−0.0431 (6)
O1	0.0787 (15)	0.0485 (12)	0.0563 (12)	0.0336 (11)	−0.0233 (11)	−0.0177 (10)
O2	0.0536 (13)	0.0670 (15)	0.0664 (14)	0.0276 (11)	−0.0103 (11)	−0.0157 (12)
O3	0.0501 (11)	0.0544 (12)	0.0470 (11)	0.0140 (9)	−0.0064 (9)	−0.0133 (9)
O4	0.0712 (15)	0.0492 (12)	0.0719 (14)	0.0318 (11)	−0.0349 (12)	−0.0278 (11)
O5	0.0585 (14)	0.0736 (16)	0.121 (3)	0.0266 (12)	−0.0419 (15)	−0.0473 (16)
O6	0.0698 (15)	0.0779 (16)	0.0975 (19)	0.0253 (13)	−0.0478 (14)	−0.0465 (15)
N1	0.0419 (12)	0.0399 (12)	0.0469 (13)	0.0165 (10)	−0.0136 (10)	−0.0108 (10)
N2	0.0472 (13)	0.0390 (12)	0.0579 (14)	0.0169 (10)	−0.0176 (11)	−0.0178 (11)
C1	0.0434 (14)	0.0378 (13)	0.0445 (14)	0.0077 (11)	−0.0132 (11)	−0.0101 (11)
C2	0.0427 (14)	0.0377 (13)	0.0486 (15)	0.0086 (11)	−0.0157 (12)	−0.0165 (11)
C3	0.0499 (15)	0.0351 (13)	0.0489 (15)	0.0107 (11)	−0.0202 (12)	−0.0155 (11)
C4	0.0640 (19)	0.0403 (14)	0.0541 (17)	0.0024 (13)	−0.0219 (14)	−0.0082 (13)
C5	0.070 (2)	0.0568 (18)	0.0477 (16)	−0.0097 (16)	−0.0160 (15)	−0.0036 (14)
C6	0.0553 (18)	0.073 (3)	0.0500 (17)	−0.0062 (16)	−0.0034 (14)	−0.0135 (15)
C7	0.0410 (15)	0.0563 (17)	0.0541 (17)	0.0060 (13)	−0.0095 (12)	−0.0153 (14)
C8	0.0478 (15)	0.0354 (13)	0.0473 (15)	0.0018 (11)	−0.0166 (12)	−0.0128 (11)
C9	0.0433 (14)	0.0390 (13)	0.0459 (14)	0.0024 (11)	−0.0140 (11)	−0.0140 (11)
C10	0.0423 (14)	0.0408 (14)	0.0508 (15)	0.0066 (11)	−0.0143 (12)	−0.0151 (12)
C11	0.0510 (18)	0.070 (2)	0.0513 (17)	0.0107 (15)	−0.0031 (14)	−0.0209 (15)
C12	0.071 (3)	0.065 (2)	0.0568 (19)	0.0050 (17)	−0.0084 (16)	−0.0161 (16)
C13	0.0484 (16)	0.0412 (14)	0.0547 (16)	0.0092 (12)	−0.0160 (13)	−0.0174 (12)
C14	0.0468 (15)	0.0405 (14)	0.0492 (15)	0.0089 (12)	−0.0147 (12)	−0.0129 (12)
C15	0.0494 (15)	0.0368 (13)	0.0441 (14)	0.0140 (11)	−0.0128 (12)	−0.0104 (11)
C16	0.0624 (18)	0.0424 (15)	0.0464 (15)	0.0067 (13)	−0.0138 (13)	−0.0149 (12)
C17	0.070 (2)	0.0594 (19)	0.0595 (19)	−0.0027 (16)	−0.0224 (16)	−0.0203 (15)
C18	0.0578 (19)	0.076 (3)	0.069 (2)	0.0039 (17)	−0.0299 (17)	−0.0203 (18)
C19	0.0485 (16)	0.0542 (17)	0.0586 (18)	0.0124 (13)	−0.0173 (14)	−0.0162 (14)
C20	0.0473 (15)	0.0372 (13)	0.0392 (13)	0.0068 (11)	−0.0082 (11)	−0.0085 (11)
C21	0.0424 (14)	0.0389 (13)	0.0436 (14)	0.0086 (11)	−0.0102 (11)	−0.0106 (11)
C22	0.0505 (17)	0.0473 (16)	0.0614 (18)	0.0093 (13)	−0.0188 (14)	−0.0155 (14)
C23	0.077 (3)	0.091 (3)	0.115 (4)	0.020 (3)	−0.054 (3)	−0.044 (3)
C24	0.102 (4)	0.101 (4)	0.108 (4)	−0.009 (3)	−0.053 (3)	−0.034 (3)

### Geometric parameters (Å, º)

**Table:**

Cl1—C7	1.731 (3)	C15—C20	1.471 (5)
Cl2—C19	1.734 (4)	C16—C17	1.356 (6)
O1—C3	1.234 (4)	C16—C20	1.410 (5)
O2—C10	1.203 (4)	C17—C18	1.394 (5)
O3—C10	1.338 (4)	C18—C19	1.371 (7)
O3—C11	1.459 (4)	C19—C21	1.398 (5)
O4—C15	1.233 (4)	C20—C21	1.403 (4)
O5—C22	1.197 (4)	C23—C24	1.434 (9)
O6—C22	1.336 (5)	N1—H1A	0.860
O6—C23	1.452 (7)	N2—H2	0.860
N1—C1	1.333 (4)	C1—H1	0.930
N1—C9	1.375 (4)	C4—H4	0.930
N2—C13	1.325 (5)	C5—H5	0.930
N2—C21	1.380 (5)	C6—H6	0.930
C1—C2	1.376 (4)	C11—H11A	0.970
C2—C3	1.448 (4)	C11—H11B	0.970
C2—C10	1.472 (4)	C12—H12A	0.960
C3—C8	1.479 (4)	C12—H12B	0.960
C4—C5	1.366 (5)	C12—H12C	0.960
C4—C8	1.406 (4)	C13—H13	0.930
C5—C6	1.386 (5)	C16—H16	0.930
C6—C7	1.374 (5)	C17—H17	0.930
C7—C9	1.412 (4)	C18—H18	0.930
C8—C9	1.393 (4)	C23—H23A	0.970
C11—C12	1.470 (5)	C23—H23B	0.970
C13—C14	1.378 (4)	C24—H24A	0.960
C14—C15	1.451 (5)	C24—H24B	0.960
C14—C22	1.465 (6)	C24—H24C	0.960
			
Cl1···N1	3.000 (3)	C9···H4^ix^	3.3960
Cl2···N2	3.015 (4)	C9···H12B^xiv^	3.3338
O1···O2	2.786 (3)	C9···H24B^x^	3.5847
O1···C1	3.593 (4)	C10···H2^iv^	3.2052
O1···C4	2.795 (4)	C10···H11B^v^	3.3254
O1···C10	2.924 (4)	C10···H16^v^	3.5387
O2···C1	3.584 (4)	C10···H17	3.5173
O2···C3	2.920 (4)	C11···H6^xvi^	3.3472
O2···C11	2.641 (4)	C11···H16^v^	3.1099
O3···C1	2.664 (4)	C11···H17	3.3049
O4···O5	2.806 (5)	C12···H6^xvi^	3.5409
O4···C13	3.591 (4)	C12···H13^viii^	3.3546
O4···C16	2.786 (5)	C12···H17	3.3672
O4···C22	2.917 (5)	C12···H24B^viii^	3.0707
O5···C13	3.579 (4)	C13···H12A^viii^	3.3475
O5···C15	2.935 (5)	C13···H17^viii^	3.4514
O5···C23	2.630 (7)	C13···H23A^xv^	3.4444
O6···C13	2.669 (5)	C13···H24A^xv^	2.9146
N1···C3	2.829 (4)	C14···H1A^vi^	3.5009
N2···C15	2.830 (4)	C14···H24A^xv^	3.0131
C1···C8	2.759 (4)	C15···H1A^vi^	2.9607
C2···C9	2.810 (4)	C15···H24A^xv^	3.4101
C4···C7	2.762 (5)	C16···H5^ix^	3.0022
C5···C9	2.787 (4)	C16···H6^ix^	3.5518
C6···C8	2.786 (4)	C16···H11A^v^	3.3311
C13···C20	2.752 (5)	C17···H5^ix^	2.9835
C14···C21	2.818 (5)	C17···H12A	3.4729
C16···C19	2.763 (5)	C18···H5^ix^	3.3207
C17···C21	2.787 (6)	C18···H18^iii^	3.2531
C18···C20	2.783 (6)	C19···H23B^i^	3.4975
C22···C24	3.574 (8)	C20···H5^ix^	3.3515
Cl1···O4^i^	3.445 (3)	C21···H16^viii^	3.5751
Cl1···O5^i^	3.539 (3)	C21···H24A^xv^	3.5848
Cl1···C22^i^	3.582 (4)	C21···H24C^xv^	3.5887
Cl2···O1^ii^	3.569 (4)	C22···H1A^vi^	3.2020
Cl2···O2^ii^	3.552 (4)	C22···H4^vii^	3.5808
Cl2···C12^iii^	3.575 (4)	C22···H12A^viii^	3.5259
O1···Cl2^iv^	3.569 (4)	C23···H4^vii^	2.9753
O1···N2^iv^	2.777 (4)	C23···H24C^xvii^	3.3501
O1···C13^iv^	3.250 (4)	C24···H4^xi^	3.4444
O2···Cl2^iv^	3.552 (4)	C24···H12C^viii^	3.5293
O2···N2^iv^	3.064 (4)	H1···Cl2^iii^	3.4520
O2···C11^v^	3.582 (6)	H1···O4^i^	2.9724
O2···C16^v^	3.411 (4)	H1···O5^i^	3.4911
O4···Cl1^vi^	3.445 (3)	H1···H5^ix^	3.5789
O4···N1^vi^	2.748 (4)	H1···H11A^xiv^	3.2713
O4···C1^vi^	3.264 (4)	H1···H12B^xiv^	3.3710
O5···Cl1^vi^	3.539 (3)	H1···H17	3.2601
O5···N1^vi^	3.035 (4)	H1A···O4^i^	1.9806
O5···C1^vi^	3.595 (4)	H1A···O5^i^	2.5061
O5···C4^vii^	3.530 (5)	H1A···C14^i^	3.5009
N1···O4^i^	2.748 (4)	H1A···C15^i^	2.9607
N1···O5^i^	3.035 (4)	H1A···C22^i^	3.2020
N2···O1^ii^	2.777 (4)	H1A···H4^ix^	3.5893
N2···O2^ii^	3.064 (4)	H1A···H11A^xiv^	3.1323
N2···C16^viii^	3.536 (4)	H1A···H12B^xiv^	3.5003
C1···O4^i^	3.264 (4)	H2···O1^ii^	2.0354
C1···O5^i^	3.595 (4)	H2···O2^ii^	2.4827
C3···C6^ix^	3.391 (6)	H2···C2^ii^	3.4993
C4···O5^vii^	3.530 (5)	H2···C3^ii^	2.9860
C4···C8^ix^	3.561 (5)	H2···C10^ii^	3.2052
C4···C9^ix^	3.320 (6)	H2···H16^viii^	3.4976
C4···C24^x^	3.394 (8)	H2···H23A^xv^	3.3595
C5···C8^ix^	3.547 (6)	H4···O5^vii^	2.8000
C5···C9^ix^	3.580 (6)	H4···N1^ix^	3.5232
C6···C3^ix^	3.391 (6)	H4···C7^ix^	3.4967
C8···C4^ix^	3.561 (5)	H4···C9^ix^	3.3960
C8···C5^ix^	3.547 (6)	H4···C22^vii^	3.5808
C9···C4^ix^	3.320 (6)	H4···C23^vii^	2.9753
C9···C5^ix^	3.580 (6)	H4···C24^x^	3.4444
C11···O2^v^	3.582 (6)	H4···H1A^ix^	3.5893
C12···Cl2^iii^	3.575 (4)	H4···H23A^vii^	2.3028
C13···O1^ii^	3.250 (4)	H4···H23B^vii^	2.8255
C13···C17^viii^	3.565 (5)	H4···H24B^x^	3.2960
C15···C18^viii^	3.496 (5)	H4···H24C^x^	2.7346
C15···C19^viii^	3.569 (4)	H5···O5^vii^	3.3522
C16···O2^v^	3.411 (4)	H5···N1^ix^	3.5029
C16···N2^viii^	3.536 (4)	H5···C1^ix^	3.3232
C16···C21^viii^	3.452 (4)	H5···C2^ix^	3.4991
C17···C13^viii^	3.565 (5)	H5···C16^ix^	3.0022
C18···C15^viii^	3.496 (5)	H5···C17^ix^	2.9835
C19···C15^viii^	3.569 (4)	H5···C18^ix^	3.3207
C20···C20^viii^	3.592 (4)	H5···C20^ix^	3.3515
C20···C21^viii^	3.555 (4)	H5···H1^ix^	3.5789
C21···C16^viii^	3.452 (4)	H5···H16^ix^	3.3282
C21···C20^viii^	3.555 (4)	H5···H17^ix^	3.2919
C22···Cl1^vi^	3.582 (4)	H5···H24C^x^	3.2924
C24···C4^xi^	3.394 (8)	H6···C3^ix^	3.4919
Cl1···H1A	2.5955	H6···C11^xii^	3.3472
Cl1···H6	2.7913	H6···C12^xii^	3.5409
Cl2···H2	2.6194	H6···C16^ix^	3.5518
Cl2···H18	2.7763	H6···H11A^xii^	3.0507
O1···H4	2.5092	H6···H11B^xii^	3.0432
O2···H11A	2.6635	H6···H12C^xii^	2.8622
O2···H11B	2.5628	H6···H16^ix^	3.2512
O3···H1	2.2987	H11A···O4^v^	2.7435
O3···H12A	2.5189	H11A···N1^xiv^	3.3055
O3···H12B	2.5684	H11A···C1^xiv^	3.3821
O3···H12C	3.2006	H11A···C16^v^	3.3311
O4···H16	2.4985	H11A···H1^xiv^	3.2713
O5···H23A	2.6999	H11A···H1A^xiv^	3.1323
O5···H23B	2.5016	H11A···H6^xvi^	3.0507
O6···H13	2.3136	H11A···H16^v^	2.4474
O6···H24A	2.5027	H11B···O2^v^	2.6385
O6···H24B	2.5533	H11B···C10^v^	3.3254
O6···H24C	3.1812	H11B···H6^xvi^	3.0432
C2···H1A	3.1484	H11B···H11B^v^	3.1324
C3···H1	3.2749	H11B···H16^v^	2.9670
C3···H4	2.6459	H11B···H17	3.1192
C4···H6	3.2247	H12A···Cl2^iii^	3.1724
C6···H4	3.2250	H12A···O6^viii^	3.0510
C7···H1A	2.5766	H12A···C13^viii^	3.3475
C7···H5	3.2255	H12A···C17	3.4729
C8···H1A	3.1641	H12A···C22^viii^	3.5259
C8···H5	3.2474	H12A···H13^viii^	2.8037
C9···H1	3.1894	H12A···H17	2.6339
C9···H4	3.2535	H12A···H24B^viii^	3.1621
C9···H6	3.2616	H12B···Cl2^iii^	3.0802
C10···H1	2.5882	H12B···N1^xiv^	3.0986
C10···H11A	2.6394	H12B···C1^xiv^	2.9743
C10···H11B	2.5927	H12B···C2^xiv^	3.0787
C14···H2	3.1480	H12B···C3^xiv^	3.3085
C15···H13	3.2727	H12B···C8^xiv^	3.4431
C15···H16	2.6415	H12B···C9^xiv^	3.3338
C16···H18	3.2230	H12B···H1^xiv^	3.3710
C18···H16	3.2258	H12B···H1A^xiv^	3.5003
C19···H2	2.5841	H12B···H24B^viii^	2.8090
C19···H17	3.2289	H12C···Cl1^xvi^	3.0035
C20···H2	3.1707	H12C···O6^viii^	3.4495
C20···H17	3.2437	H12C···C24^viii^	3.5293
C21···H13	3.1862	H12C···H6^xvi^	2.8622
C21···H16	3.2643	H12C···H13^viii^	3.0454
C21···H18	3.2495	H12C···H24B^viii^	2.7388
C22···H13	2.5883	H13···O1^ii^	2.9311
C22···H23A	2.6455	H13···O2^ii^	3.4981
C22···H23B	2.5678	H13···C12^viii^	3.3546
H1···H1A	2.1951	H13···H12A^viii^	2.8037
H2···H13	2.1819	H13···H12C^viii^	3.0454
H4···H5	2.2876	H13···H17^viii^	3.5783
H5···H6	2.3133	H13···H23A^xv^	3.2242
H11A···H12A	2.7948	H13···H24A^xv^	3.1982
H11A···H12B	2.2943	H16···O2^v^	2.6581
H11A···H12C	2.3285	H16···N2^viii^	3.4800
H11B···H12A	2.3300	H16···C10^v^	3.5387
H11B···H12B	2.7947	H16···C11^v^	3.1099
H11B···H12C	2.2929	H16···C21^viii^	3.5751
H16···H17	2.2784	H16···H2^viii^	3.4976
H17···H18	2.3222	H16···H5^ix^	3.3282
H23A···H24A	2.2952	H16···H6^ix^	3.2512
H23A···H24B	2.7647	H16···H11A^v^	2.4474
H23A···H24C	2.2536	H16···H11B^v^	2.9670
H23B···H24A	2.7650	H17···O2^v^	3.5157
H23B···H24B	2.2578	H17···O3	2.9058
H23B···H24C	2.2908	H17···C10	3.5173
Cl1···H12C^xii^	3.0035	H17···C11	3.3049
Cl1···H24A^xiii^	3.0728	H17···C12	3.3672
Cl1···H24B^xiii^	3.4138	H17···C13^viii^	3.4514
Cl2···H1^iii^	3.4520	H17···H1	3.2601
Cl2···H12A^iii^	3.1724	H17···H5^ix^	3.2919
Cl2···H12B^iii^	3.0802	H17···H11B	3.1192
Cl2···H23B^i^	3.1372	H17···H12A	2.6339
O1···H2^iv^	2.0354	H17···H13^viii^	3.5783
O1···H13^iv^	2.9311	H18···O5^i^	3.2961
O1···H23A^vii^	2.7404	H18···C18^iii^	3.2531
O2···H2^iv^	2.4827	H18···H18^iii^	2.3421
O2···H11B^v^	2.6385	H23A···O1^vii^	2.7404
O2···H13^iv^	3.4981	H23A···N2^xv^	3.5095
O2···H16^v^	2.6581	H23A···C4^vii^	3.1923
O2···H17^v^	3.5157	H23A···C13^xv^	3.4444
O3···H17	2.9058	H23A···H2^xv^	3.3595
O4···H1^vi^	2.9724	H23A···H4^vii^	2.3028
O4···H1A^vi^	1.9806	H23A···H13^xv^	3.2242
O4···H11A^v^	2.7435	H23A···H24C^xvii^	3.1658
O5···H1^vi^	3.4911	H23B···Cl2^vi^	3.1372
O5···H1A^vi^	2.5061	H23B···C19^vi^	3.4975
O5···H4^vii^	2.8000	H23B···H4^vii^	2.8255
O5···H5^vii^	3.3522	H23B···H24C^xvii^	2.7935
O5···H18^vi^	3.2961	H24A···Cl1^xiii^	3.0728
O6···H12A^viii^	3.0510	H24A···N2^xv^	3.2174
O6···H12C^viii^	3.4495	H24A···C13^xv^	2.9146
N1···H4^ix^	3.5232	H24A···C14^xv^	3.0131
N1···H5^ix^	3.5029	H24A···C15^xv^	3.4101
N1···H11A^xiv^	3.3055	H24A···C21^xv^	3.5848
N1···H12B^xiv^	3.0986	H24A···H13^xv^	3.1982
N2···H16^viii^	3.4800	H24B···Cl1^xiii^	3.4138
N2···H23A^xv^	3.5095	H24B···C3^xi^	3.5623
N2···H24A^xv^	3.2174	H24B···C4^xi^	3.0695
N2···H24C^xv^	3.4565	H24B···C5^xi^	3.4891
C1···H5^ix^	3.3232	H24B···C8^xi^	3.1021
C1···H11A^xiv^	3.3821	H24B···C9^xi^	3.5847
C1···H12B^xiv^	2.9743	H24B···C12^viii^	3.0707
C2···H2^iv^	3.4993	H24B···H4^xi^	3.2960
C2···H5^ix^	3.4991	H24B···H12A^viii^	3.1621
C2···H12B^xiv^	3.0787	H24B···H12B^viii^	2.8090
C3···H2^iv^	2.9860	H24B···H12C^viii^	2.7388
C3···H6^ix^	3.4919	H24C···N2^xv^	3.4565
C3···H12B^xiv^	3.3085	H24C···C4^xi^	2.8703
C3···H24B^x^	3.5623	H24C···C5^xi^	3.2032
C4···H23A^vii^	3.1923	H24C···C8^xi^	3.5332
C4···H24B^x^	3.0695	H24C···C21^xv^	3.5887
C4···H24C^x^	2.8703	H24C···C23^xvii^	3.3501
C5···H24B^x^	3.4891	H24C···H4^xi^	2.7346
C5···H24C^x^	3.2032	H24C···H5^xi^	3.2924
C7···H4^ix^	3.4967	H24C···H23A^xvii^	3.1658
C8···H12B^xiv^	3.4431	H24C···H23B^xvii^	2.7935
C8···H24B^x^	3.1021	H24C···H24C^xvii^	3.2614
C8···H24C^x^	3.5332		
			
C10—O3—C11	116.0 (3)	O5—C22—O6	122.1 (4)
C22—O6—C23	116.2 (3)	O5—C22—C14	126.0 (4)
C1—N1—C9	121.5 (3)	O6—C22—C14	111.8 (3)
C13—N2—C21	121.6 (3)	O6—C23—C24	108.7 (4)
N1—C1—C2	123.5 (3)	C1—N1—H1A	119.268
C1—C2—C3	119.6 (3)	C9—N1—H1A	119.263
C1—C2—C10	119.8 (3)	C13—N2—H2	119.225
C3—C2—C10	120.7 (3)	C21—N2—H2	119.224
O1—C3—C2	125.2 (3)	N1—C1—H1	118.267
O1—C3—C8	119.6 (3)	C2—C1—H1	118.268
C2—C3—C8	115.1 (3)	C5—C4—H4	119.631
C5—C4—C8	120.7 (3)	C8—C4—H4	119.632
C4—C5—C6	120.4 (3)	C4—C5—H5	119.785
C5—C6—C7	119.9 (3)	C6—C5—H5	119.782
Cl1—C7—C6	119.9 (3)	C5—C6—H6	120.055
Cl1—C7—C9	119.3 (3)	C7—C6—H6	120.052
C6—C7—C9	120.8 (3)	O3—C11—H11A	110.210
C3—C8—C4	119.9 (3)	O3—C11—H11B	110.206
C3—C8—C9	120.7 (3)	C12—C11—H11A	110.205
C4—C8—C9	119.3 (3)	C12—C11—H11B	110.201
N1—C9—C7	121.7 (3)	H11A—C11—H11B	108.495
N1—C9—C8	119.6 (3)	C11—C12—H12A	109.473
C7—C9—C8	118.8 (3)	C11—C12—H12B	109.470
O2—C10—O3	122.5 (3)	C11—C12—H12C	109.478
O2—C10—C2	125.3 (3)	H12A—C12—H12B	109.463
O3—C10—C2	112.2 (3)	H12A—C12—H12C	109.472
O3—C11—C12	107.5 (3)	H12B—C12—H12C	109.471
N2—C13—C14	124.1 (4)	N2—C13—H13	117.980
C13—C14—C15	119.1 (4)	C14—C13—H13	117.965
C13—C14—C22	120.1 (4)	C17—C16—H16	119.658
C15—C14—C22	120.8 (3)	C20—C16—H16	119.643
O4—C15—C14	125.0 (4)	C16—C17—H17	119.773
O4—C15—C20	119.7 (4)	C18—C17—H17	119.776
C14—C15—C20	115.3 (3)	C17—C18—H18	120.092
C17—C16—C20	120.7 (3)	C19—C18—H18	120.094
C16—C17—C18	120.5 (4)	O6—C23—H23A	109.956
C17—C18—C19	119.8 (4)	O6—C23—H23B	109.955
Cl2—C19—C18	119.2 (3)	C24—C23—H23A	109.957
Cl2—C19—C21	119.7 (3)	C24—C23—H23B	109.953
C18—C19—C21	121.1 (3)	H23A—C23—H23B	108.342
C15—C20—C16	119.7 (3)	C23—C24—H24A	109.475
C15—C20—C21	121.1 (3)	C23—C24—H24B	109.472
C16—C20—C21	119.1 (3)	C23—C24—H24C	109.473
N2—C21—C19	122.4 (3)	H24A—C24—H24B	109.472
N2—C21—C20	118.9 (3)	H24A—C24—H24C	109.470
C19—C21—C20	118.8 (4)	H24B—C24—H24C	109.466
			
C10—O3—C11—C12	−176.3 (3)	C3—C8—C9—N1	−2.5 (5)
C10—O3—C11—H11A	63.6	C3—C8—C9—C7	177.9 (3)
C10—O3—C11—H11B	−56.2	C4—C8—C9—N1	177.7 (3)
C11—O3—C10—O2	1.3 (5)	C4—C8—C9—C7	−1.9 (5)
C11—O3—C10—C2	−178.9 (3)	O3—C11—C12—H12A	56.7
C22—O6—C23—C24	173.8 (3)	O3—C11—C12—H12B	−63.3
C22—O6—C23—H23A	−65.8	O3—C11—C12—H12C	176.7
C22—O6—C23—H23B	53.4	H11A—C11—C12—H12A	176.8
C23—O6—C22—O5	−5.1 (4)	H11A—C11—C12—H12B	56.8
C23—O6—C22—C14	173.6 (3)	H11A—C11—C12—H12C	−63.2
C1—N1—C9—C7	179.6 (3)	H11B—C11—C12—H12A	−63.5
C1—N1—C9—C8	0.0 (5)	H11B—C11—C12—H12B	176.6
C9—N1—C1—C2	1.2 (5)	H11B—C11—C12—H12C	56.5
C9—N1—C1—H1	−178.8	N2—C13—C14—C15	−1.0 (4)
H1A—N1—C1—C2	−178.8	N2—C13—C14—C22	179.8 (2)
H1A—N1—C1—H1	1.2	H13—C13—C14—C15	179.0
H1A—N1—C9—C7	−0.4	H13—C13—C14—C22	−0.2
H1A—N1—C9—C8	−180.0	C13—C14—C15—O4	−177.9 (3)
C13—N2—C21—C19	179.0 (2)	C13—C14—C15—C20	1.0 (4)
C13—N2—C21—C20	−0.4 (4)	C13—C14—C22—O5	−168.2 (3)
C21—N2—C13—C14	0.7 (4)	C13—C14—C22—O6	13.1 (4)
C21—N2—C13—H13	−179.3	C15—C14—C22—O5	12.6 (4)
H2—N2—C13—C14	−179.3	C15—C14—C22—O6	−166.0 (2)
H2—N2—C13—H13	0.7	C22—C14—C15—O4	1.4 (4)
H2—N2—C21—C19	−1.0	C22—C14—C15—C20	−179.8 (2)
H2—N2—C21—C20	179.6	O4—C15—C20—C16	−1.9 (4)
N1—C1—C2—C3	0.2 (5)	O4—C15—C20—C21	178.2 (2)
N1—C1—C2—C10	−179.2 (3)	C14—C15—C20—C16	179.21 (19)
H1—C1—C2—C3	−179.8	C14—C15—C20—C21	−0.7 (3)
H1—C1—C2—C10	0.8	C17—C16—C20—C15	178.5 (3)
C1—C2—C3—O1	176.1 (3)	C17—C16—C20—C21	−1.6 (4)
C1—C2—C3—C8	−2.5 (5)	C20—C16—C17—C18	0.7 (4)
C1—C2—C10—O2	173.2 (4)	C20—C16—C17—H17	−179.3
C1—C2—C10—O3	−6.5 (5)	H16—C16—C17—C18	−179.3
C3—C2—C10—O2	−6.2 (6)	H16—C16—C17—H17	0.7
C3—C2—C10—O3	174.1 (3)	H16—C16—C20—C15	−1.5
C10—C2—C3—O1	−4.5 (6)	H16—C16—C20—C21	178.4
C10—C2—C3—C8	176.9 (3)	C16—C17—C18—C19	0.8 (5)
O1—C3—C8—C4	4.8 (5)	C16—C17—C18—H18	−179.2
O1—C3—C8—C9	−175.0 (3)	H17—C17—C18—C19	−179.2
C2—C3—C8—C4	−176.5 (3)	H17—C17—C18—H18	0.8
C2—C3—C8—C9	3.6 (5)	C17—C18—C19—Cl2	178.0 (3)
C5—C4—C8—C3	−178.1 (4)	C17—C18—C19—C21	−1.3 (5)
C5—C4—C8—C9	1.7 (6)	H18—C18—C19—Cl2	−2.0
C8—C4—C5—C6	−0.4 (6)	H18—C18—C19—C21	178.7
C8—C4—C5—H5	179.6	Cl2—C19—C21—N2	1.6 (4)
H4—C4—C5—C6	179.6	Cl2—C19—C21—C20	−179.00 (16)
H4—C4—C5—H5	−0.4	C18—C19—C21—N2	−179.0 (3)
H4—C4—C8—C3	1.9	C18—C19—C21—C20	0.4 (4)
H4—C4—C8—C9	−178.2	C15—C20—C21—N2	0.4 (4)
C4—C5—C6—C7	−0.7 (7)	C15—C20—C21—C19	−178.99 (19)
C4—C5—C6—H6	179.3	C16—C20—C21—N2	−179.5 (2)
H5—C5—C6—C7	179.3	C16—C20—C21—C19	1.1 (4)
H5—C5—C6—H6	−0.7	O6—C23—C24—H24A	56.6
C5—C6—C7—Cl1	−178.8 (4)	O6—C23—C24—H24B	−63.4
C5—C6—C7—C9	0.5 (7)	O6—C23—C24—H24C	176.6
H6—C6—C7—Cl1	1.2	H23A—C23—C24—H24A	−63.8
H6—C6—C7—C9	−179.5	H23A—C23—C24—H24B	176.2
Cl1—C7—C9—N1	0.6 (5)	H23A—C23—C24—H24C	56.2
Cl1—C7—C9—C8	−179.8 (3)	H23B—C23—C24—H24A	177.0
C6—C7—C9—N1	−178.7 (4)	H23B—C23—C24—H24B	57.0
C6—C7—C9—C8	0.9 (6)	H23B—C23—C24—H24C	−63.0

Symmetry codes: (i) *x*−1, *y*, *z*; (ii) *x*, *y*+1, *z*; (iii) −*x*+1, −*y*+2, −*z*+1; (iv) *x*, *y*−1, *z*; (v) −*x*+2, −*y*+1, −*z*+1; (vi) *x*+1, *y*, *z*; (vii) −*x*+2, −*y*+1, −*z*+2; (viii) −*x*+2, −*y*+2, −*z*+1; (ix) −*x*+1, −*y*+1, −*z*+2; (x) *x*−1, *y*−1, *z*; (xi) *x*+1, *y*+1, *z*; (xii) *x*−1, *y*, *z*+1; (xiii) −*x*+1, −*y*+2, −*z*+2; (xiv) −*x*+1, −*y*+1, −*z*+1; (xv) −*x*+2, −*y*+2, −*z*+2; (xvi) *x*+1, *y*, *z*−1; (xvii) −*x*+3, −*y*+2, −*z*+2.

### Hydrogen-bond geometry (Å, º)

**Table:**

*D*—H···*A*	*D*—H	H···*A*	*D*···*A*	*D*—H···*A*
N1—H1*A*···O4^i^	0.86	1.98	2.748 (4)	148
N1—H1*A*···O5^i^	0.86	2.51	3.035 (4)	121
N2—H2···O1^ii^	0.86	2.04	2.777 (4)	144
N2—H2···O2^ii^	0.86	2.48	3.064 (4)	126

Symmetry codes: (i) *x*−1, *y*, *z*; (ii) *x*, *y*+1, *z*.
