# Supplementary figures and images for: Crystal structure of ethyl 8-chloro-4-oxo-1,4-di­hydro­quinoline-3-carboxyl­ate
Source: Acta Crystallogr E Crystallogr Commun. 2015 Jul 15;71(Pt 8):o566. doi: 10.1107/S2056989015013171 (PMC4571399; doi:10.1107/S2056989015013171)

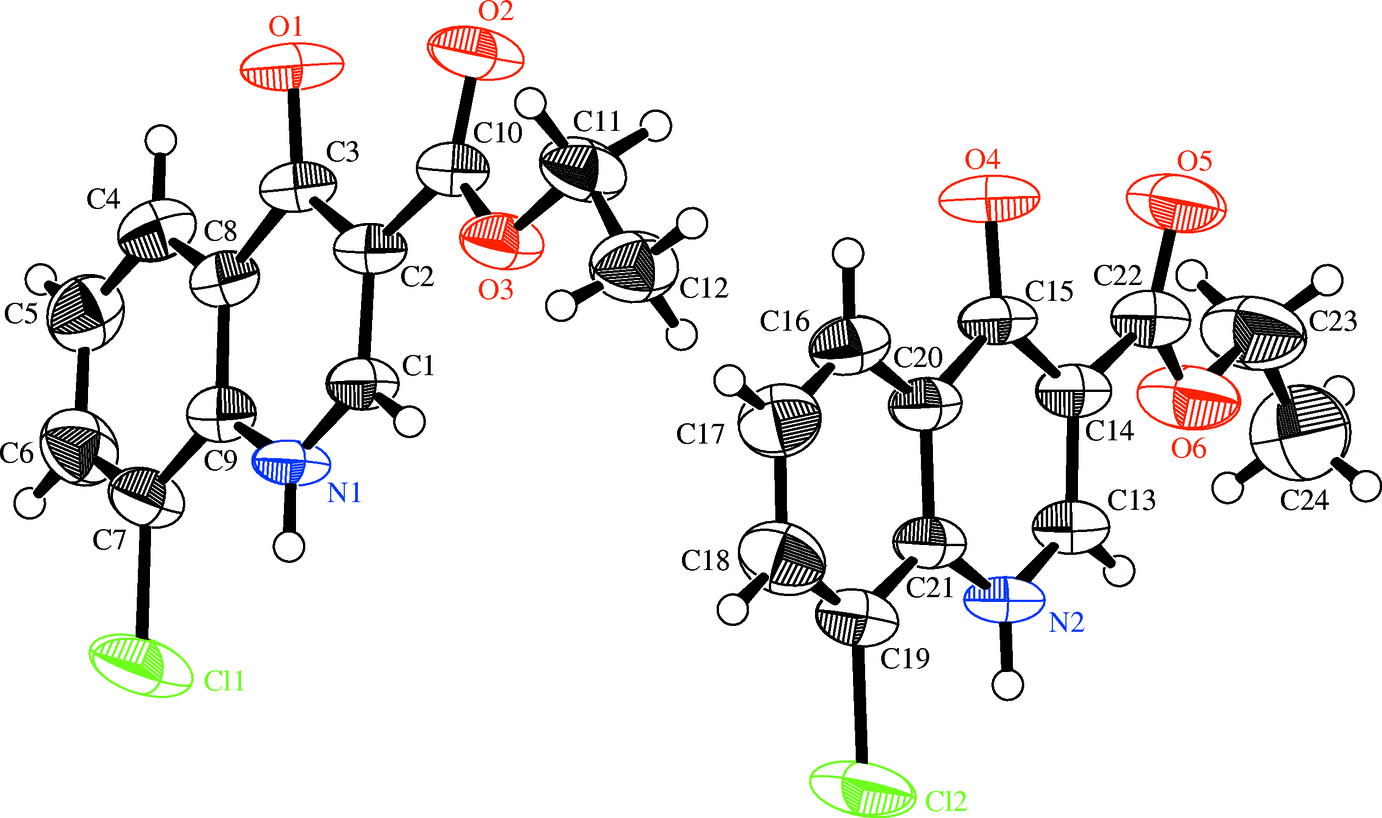

Supplement: Supplementary file 4 [file e-71-0o566-fig1.tif]

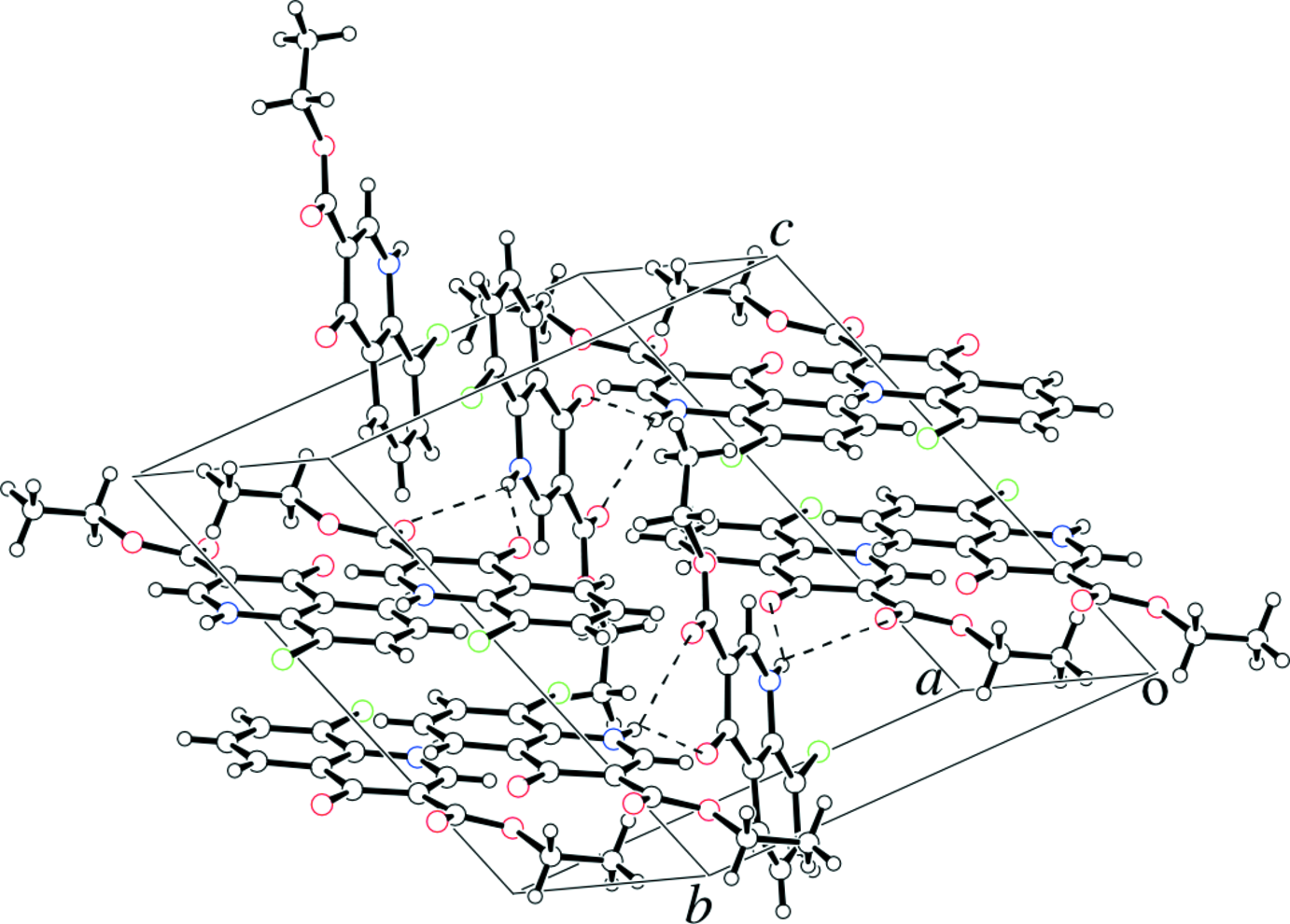

Supplement: Supplementary file 5 [file e-71-0o566-fig2.tif]
